# A case report revealing acute onset psychosis and cognitive impairment as primary manifestation in relapsing‐remitting multiple sclerosis

**DOI:** 10.1002/ccr3.2781

**Published:** 2020-03-04

**Authors:** Anirban Nandy, Michael Nielsen, Claudia Hilt, Poul Henning Mogensen, Yousef Yavarian

**Affiliations:** ^1^ Department of Neurology Aalborg University Hospital Aalborg Denmark; ^2^ Department of Radiology Aalborg University Hospital Aalborg Denmark

**Keywords:** acute psychosis, atypical RRMS, cognitive impairment, early MS presentation

## Abstract

Acute psychosis and cognitive impairment is a significant problem in RRMS. As it concerns in relatively young age group, our case report underscores the importance of early recognition which could impose diagnostic challenge in multiple sclerosis.

## Graphical abstract

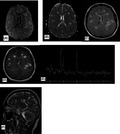

## 1 INTRODUCTION

Multiple sclerosis (MS) is a demyelinating disorder of the central nervous system. Neuropsychiatric symptoms have previously been reported as a rare manifestation of MS,^1^ yet onset of MS with psychosis is rarely encountered especially with relapsing‐remitting type of multiple sclerosis (RRMS). Untreated psychosis in patients with MS can adversely impact on MS medication, levels of disability, and quality of life.^1^ A 23‐year‐old Caucasian male was admitted due to sudden onset of cognitive deficits, agitation, aggressive self‐harming behavior, and neurological symptoms with paresis of the right upper extremity along with ataxic gait. His clinical, radiological, and laboratorial examinations initially lead to the suspicion of ADEM, eventually diagnosed with RRMS. Acute onset of neuropsychotic symptoms with MRI brain verified fulminant contrast enhancing ovoid lesions, both nodular and ring‐enhancing involving both cerebral hemispheres, response to high dose of steroids and plasma exchange, with a relatively short interval between psychiatric and neurological signs indicate a high likelihood that acute psychosis in our patient could be a manifestation of underlying MS.

## 2 CASE REPORT

A 23‐year‐old previously healthy Caucasian male, university student with no previous psychiatric history, was hospitalized with a subacute onset, four‐day anamnesis of confusion, extreme restlessness, and diffuse cognitive deficits. Due to aggressive self‐harming behavior, paranoia, suicidal thoughts, and motor restlessness, psychiatric consultation was requested for assistance. There was no history of drug or alcohol abuse, and no known family history of mood or psychotic disorder.

A general examination revealed no rashes and normal vital parameters. Physical examination showed decreased motor function of the right lower extremity along with ataxic gait without any signs of an underlying myopathy. He was awake but remained confused, agitated, restless, had agnosia, and was not able to recognize either his mother or girlfriend. During admission, the patient expressed unrealistic and irrelevant thoughts. Furthermore, increased latency to give his personal data such as birth‐date was observed. He was aggressive with suicidal thoughts. These symptoms had dramatically worsened within 2 days prior to admission. There were no associated seizures or headaches. He was afebrile (37.1°) with peripheral oxygen saturation 98%, blood pressure (135/67 mmHg), respiratory rate (21 breaths/min), and heart rate (83 beats/min). The initial serum biochemistry showed results within normal parameters for hematology, electrolytes, infection parameters, and thyroid test (including thyroid antibodies**).** Acute routine cerebrospinal fluid examination revealed 5 leukocytes (mononuclear), lactate 1.6 mmol/L, mildly elevated protein 0.56 g/L and glucose 3.6 mmol/L, normal IgG, and negative oligoclonal bands. Intrathecal Herpes simplex virus, Varicella zoster, and Borrelia PCRs were negative. Cytomegalovirus IgG was also negative.

**Figure 1:**
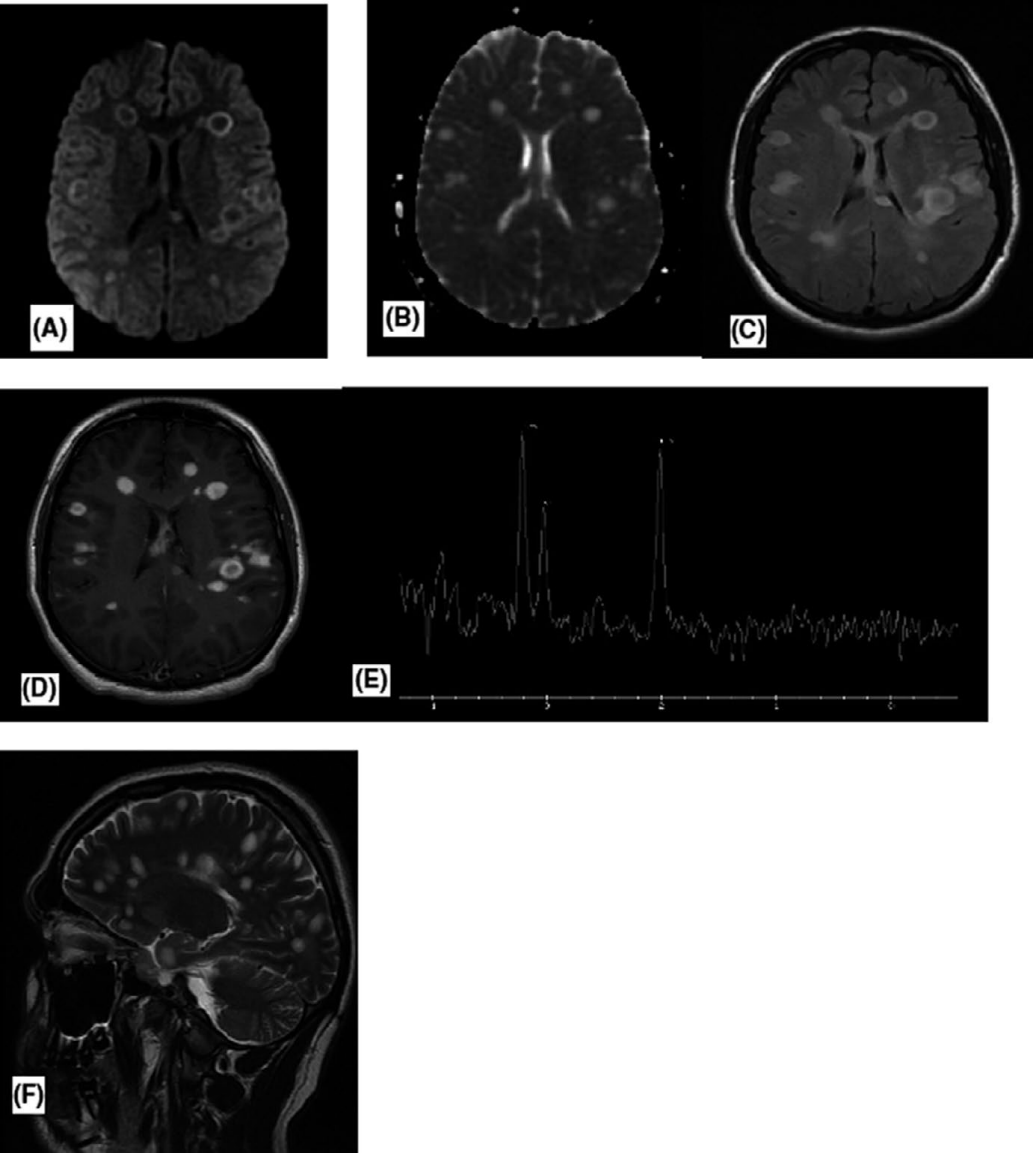
Axial DWI (A) and corresponding ADC—map (B) show multiple MS plaques with peripheral high signal on DWI and T2 shine through on the ADC. On axial T2 flair (C) multiple high‐signal intensity plaques are seen and on axial T1 + contrast (D) the vast majority of the plaques show ring enhancement. Spectroscopy (E) shows increased choline and almost normal NAA. Sagittal T2 (F) shows multiple MS plaques

CT‐cerebrum showed multiple low‐density lesions without intracranial hemorrhages, ischemia or space‐occupying lesions. MRI brain showed fulminant contrast enhancing ovoid lesions, both nodular and ring‐enhancing involving both cerebral hemispheres involving callososeptal interface, also including left infratentorial involving pons and medulla oblongata raising suspicion of severe demyelination disorders (eg, acute disseminated encephalomyelitis (ADEM) or MS), malignancy and neuroinfection (Figure 1). No medullary lesions were found (Figure 3).

Further blood test revealed negative Quantiferon TB test, toxoplasma serology, HIV 1 + 2 and viral hepatitis screening. Control cerebrospinal fluid analysis 5 weeks after the initial presentation showed leukocytes 3 (mononuclear), lactate 1.7 mmol/L, protein 0.55 g/L, glucose 3.4 mmol/L, high IgG ratio, and positive oligoclonal bands. Serology for autoimmune synaptic encephalitis was negative. Analysis of cerebrospinal fluid for markers of cancer and computer tomography of thorax and abdomen did not shown signs of malignancy.

ECG showed sinus rhythm with frequency 80/min without ST segment deviation. Transthoracic echocardiography showed physiological valve functions with ejection fraction over 60 mL.

Neuropsychological evaluation revealed mini‐mental state examination (MMSE) 25, with decreased executive function, processing speed, and associations. In conclusion, cognitive reduction due to organic pathology.

**Figure 2:**
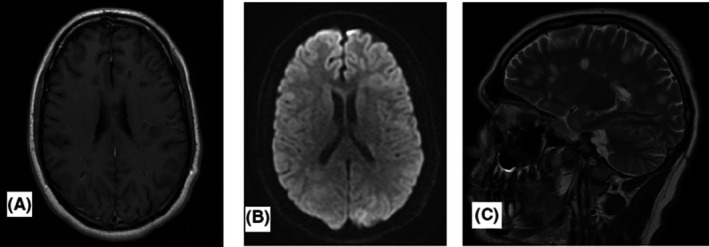
Control scanning axial T1 + control (a) and DWI (b) show the regression of contrast enhancement and high‐signal intensity in general. Sagital T2 (c) shows still multiple MS plaques

Control MRI brain after 5 weeks from the first scan showed partial regression of the lesion load with significant reduction of contrast enhancement and DWI signals (Figure 2). The patient treated with high‐dose intravenous methylprednisolone and 6 cycles of plasmapheresis between the two MRI brain scans. Follow‐up of 6 weeks after symptom onset showed near normal cognitive and physical function. No sensory or motor deficit was found on examination. The psychiatric symptoms had resolved completely. He reported slight concentration difficulties and tiredness. Immunomodulatory treatment with Cladribine had been initiated with further follow‐up at MS outpatient clinic.

## 3 DISCUSSION

**Figure 3:**
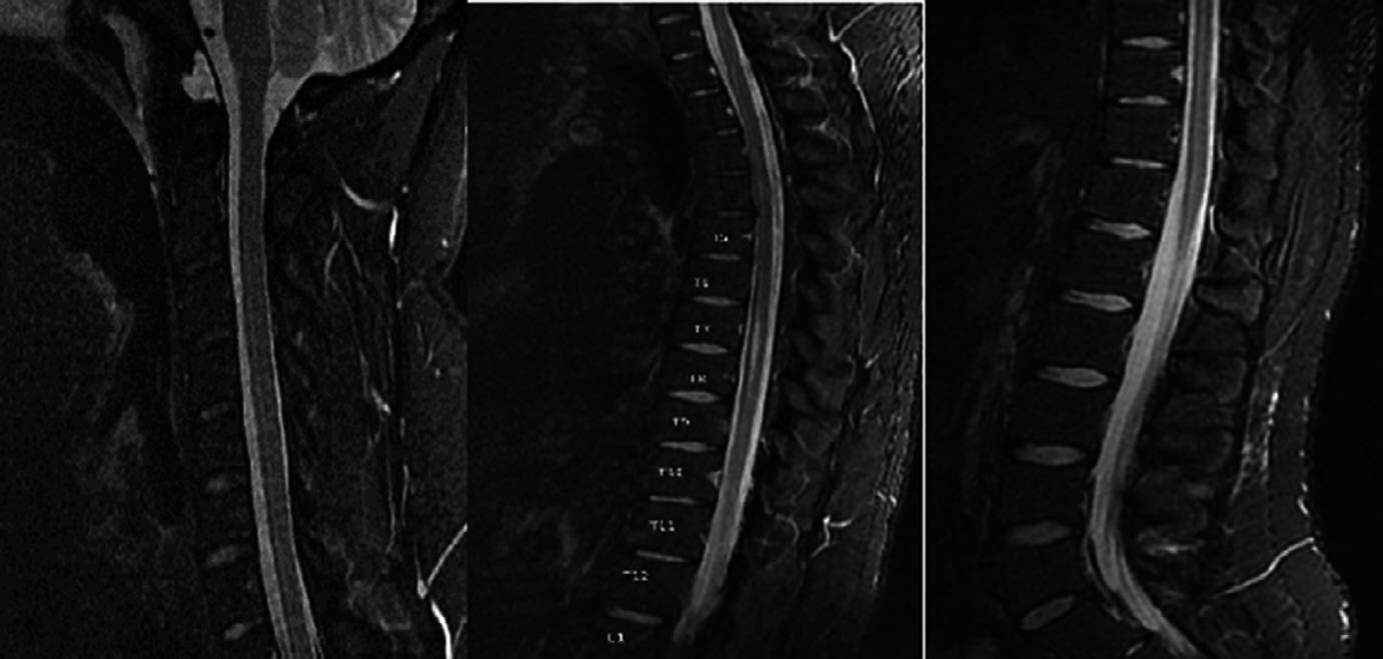
During admission, contrast MRI of cervicothoracic STIR sequences did not show any medullary pathology

Neuroadiologically and clinically ADEM seems less likely due to involvement of callososeptal interface and lack of previous infection or vaccination in anamnesis. Multifocal lymphoma and primary brain tumor were also less likely due to atypical localization, and because there was only one lesion with diffusion restriction, but all other lesions were with T2 shine through phenomenon (Figure 1A, B, and F). In addition, perfusion scan was performed, which did not show increased perfusion. Brain spectroscopy showed increased choline, but almost normal N‐ acetyl‐aspartate (NAA), which points away from tumor and lymphomas (Figure 1E). Spectroscopic studies in MS typically show increased choline and normal NAA, which was the case with our patient.^2^, ^3^ In literature, increased lipid and lactate has also been described,^4^ which we have not found in this patient, perhaps because it depends on which MS phase spectroscopy been executed. No medullary lesions were revealed (Figure 3).

The collected information narrowed the list of differential diagnosis between demyelinating diseases. A control cerebrospinal fluid investigation after 5 weeks showed high IgG index and oligoclonal bands, suggesting the probable diagnosis of relapsing‐remitting multiple sclerosis (RRMS). Control MRI scan showed clear regression of contrast enhancement on T1 + contrast and signal on DWI sequences (Figure 2A,B), but MS plaques were still high signaling on T2‐weighted sequence (Figure 2C).

Psychosis in multiple sclerosis considered as a rare onset manifestation.^1^, ^5^ Cognitive impairment is part of the clinical spectrum of multiple sclerosis (MS). Depending on the disease phase and type, 40%‐65% of MS patients develop various degrees of cognitive dysfunction. Prevalence rates of psychosis in MS are two three times higher than in the general population.^5^ Our patient was a young male who presented with acute psychotic symptoms with focal neurological deficits. There was no previous history of any diseases. The MRI brain and spinal cord with contrast showed intense and fulminant attack of inflammation with nodular ring‐enhancing lesions in both cerebral hemispheres without affecting the spinal cord, raised suspicion of ADEM, infection or demyelinating disorder. Even though initial cerebrospinal fluid investigation was normal, a new cerebrospinal fluid study after 5 weeks revealed positive oligoclonal bands and increased IgG index, ultimately diagnosing disseminated sclerosis of relapsing‐remitting variant.

In the MAGNIMS study, 63 out of 191 patients with progressive MS were diagnosed with cognitive impairment (CI) (28.6%).^6^ In one study, cognitive impairment was diagnosed in 56.5% out of 23 PPMS patients.^7^ The larger Dutch study found that cognitive impairment was more severe in PPMS and SPMS than in RRMS.^8^ The prevalence of neuropsychiatric symptoms in MS can vary according to various series, and some authors report levels as high as 95%.^6^, ^7^ Depressive symptoms are most frequent among these. Psychotic symptoms are rare as initial findings of MS.^9^ The relationship between cognitive impairment and subcortical white matter pathology has been reported to be associate with significant periventricular lesion burdens, especially in the temporal region.^10^ This finding helps in understanding the biological basis for psychosis and brain areas involved in psychosis. Over the last decade, an increasing number of observations have provided evidence of a primary role of cortical pathology—that is, inflammatory focal lesions (cortical lesions), atrophy and cortical thickness—in determining global and selective cognitive disability in MS.^11^, ^12^

Our patient fulfills the criteria for MS since oligoclonal bands were present with positive MRI cerebrum findings. He was eventually started on prophylactic Cladribine therapy as a second‐line treatment for MS.

Even though the patient continued to be mildly affected cognitively 4‐5 weeks after the onset of symptoms, plasmapheresis along with atypical antipsychotic medications gave excellent result against his psychosis.

## 4 CONCLUSION

In conclusion, acute onset psychosis and cognitive impairment is a significant problem in RRMS. Particularly in RRMS, the incidence of psychosis and CI is approximately 40%, involving complex attention, processing speed and memory and executive dysfunction, agitation, and self‐harming behavior. As RRMS in relatively young age group, shown worsening of cognitive dysfunction with psychosis, our case report underscores the importance of early recognition of acute psychosis and cognitive impairment, which could impose a diagnostic challenge in multiple sclerosis.

## CONFLICT OF INTEREST

None declared.

## AUTHOR CONTRIBUTIONS

AN: involved in clinical follow‐up and wrote the manuscript. CH: diagnosed the patient and involved in clinical follow‐up. YY: involved in reporting MRI scans and contributed the radiological aspects. MN: involved in the diagnosis and valuable contribution in the manuscript. PM: involved in the diagnosis and valuable contribution in the manuscript.
